# Spiritualism

**Published:** 1857-02

**Authors:** 


### SPIRITUALISM.

CHATTANOOGA, TENN., *July* 14*th*, 1856.

You have doubtless seen in the public prints the new exposition of Spiritualism, under the term of "Volaurology." Some knowledge gathered of its author, will enable me to give information of interest to your readers.

The Rev. J. H, Hall is about thirty-two years of age, five feet, five or six inches; a resident of this State, and native of Kentucky—has been living in retired life for some years past, owing to an affection of the eyes, which prevented him from engaging in clerical duties. At one time he was the editor of a religious Monthly published in Philadelphia, subsequently owner and partial editor of the *Christian Union and Religious Memorial*, New York. Of late years he was associated as editor of a Southern literary newspaper, and correspondent of the *Southern Parlor Magazine*, published at Memphis. He founded a flourishing female school in East Tennessee, which he gave up for the principalship of a similar institution in New York City. Of a natural disposition to search into the marvellous, and a votary of science in every sense of the word, the Spiritual Manifestation, so-called, engaged his attention from the beginning. It is a curious circumstance to note what different results scientific minds reach after the investigation of the spiritual problem. Judge Edmonds, Profs. Hare, Dexter, and others, on one side, and Dods, Mahan and Hall on the other. I do not know much about the theories of those gentlemen, excepting that of Mr. Hall. He takes the ground that the supposed *spirit's power is a physical emanation—traces it to its seat in the human body, and shows where it exists, and how to neutralize its effects, or paralyse its power immediately.* If this is not showing that he understands his subject, I profess not to be able to know what demonstration is. While the religious journals ascribe the agency to the Devil, and the secular think it a sort of sleight-of hand, the scientific world *disputes* the facts as occurring. Mr. Hall comes out fairly and boldly, acknowledges the strange manifestations, but shows them to proceed from the very constitution of our being. How much more rational and gratifying is this, than the presumptive admission of supernatural agency. Hall says like a philosopher—"God once worked by miracle, but this is an age in which he works by science." He is familiar with his subject. When the Rapping first appeared —before Judge Edmonds' advocacy of Spiritualism; before any spiritual newspapers were printed; before the editors of those spiritual newspapers appeared in public—he was an investigator of the "Stratford Mysteries." This argues much in his behalf for the strength of his mind and the reputation of his name. As an example of his method of "searching the spirits," he enclosed a letter to a celebrated spiritualist to describe the author. According to request, *it was done.* A very minute description was given, and the letter returned He then requested the spirits to describe the character of the writer of another letter, seal unopened. This the spirits did. The descriptions were different, and neither of them *correct;* besides this, the first and second letter was one and the same. He wrote to the spiritualist to know whether one or more spirits had given that examination, to which the reverend spiritualist replied, that it was done by a "convocation of spirits, and a president of them." I mention this circumstance, because the parties are men in high life and reputation, and what may be affirmed of one, *Disce Omnes.*

I would not have your readers understand that Mr. Hall believes in the power of describing authors by their hand-writing inclosed in an envelope; on the contrary, he shows how it is done; but he feels indignant that intelligent public leaders of the day, pretending to set themselves up as such, should pervert the truth into a falsehood. He says of the strange mysteries witnessed by him in the families of Judge Edmonds, Foxes, Concklin and others, he finds a counterpart or more than equivalent in the medical records of past time. Davis, Jay, Harris, and other clairvoyants, are not exceptions of power attainable by every one. If they chose to do it they can be placed upon a parallel with them in respect to their extraordinary mental development. He does not deny the power of healing, but shows that it is an element of our nature. To ascribe it to spiritual agency is extremely superstitious. In the management of the Tables, which a New York paper called the *Evangelist* says, has certainly "the devil in it,"—the Volaurologist says, "Then all men, women and children are devils," for he shows them how they can do it. I would not name the principles of the new science with the view of forestalling the exposition of the author, for he will doubtless visit your city in its turn; but I am prepared to say, that I consider the explanation of Spiritualism a perfect one, without any dodging. I am not unfriendly to its claims, but those strange things are accounted for, and when that is done by natural science, supported by well known principles, why should we seek the solution in the hobgoblins of hydra-headed Spiritualism? G. B.

The above letter was cut from a stray newspaper last Summer. Our attention was recalled to it by an editorial in the *Medical Gazette* of this city. The social and professional position of its learned editor demands a respectful consideration of his opinions. He says, in the January number, page seventeen:

"We mean to be understood, when we affirm, that it is *not true* in any case, that tables or chairs, or any other body whose specific gravity is greater than air, has been moved at all without physical force, and we care not how many 'physicians, clergymen,' &c., are among the witnesses."

There are few medical men whose information is as extensive as that of Dr. Reese. But in the discussion of any subject where the testimony of several persons is to be taken, upon which to make a decision, the statement of one as to a fact is as good as that of another, supposing that all are of ordinary intelligence. Hence a mere contradiction carries with it no conclusiveness. One of the Beecher daughters says, that she "*rode around a room on a table, touched by nothing but the fingers of a delicate girl*, who went around the room with the table, just touching it with her finger." Dr. Reese does not believe this statement. But Miss Beecher was present. She was part of the fact, and we are bound to believe her testimony, or that she was miserably deceived. Many persons measure their wisdom by the amount of incredulity which they can exhibit. Dr. Reese is wholly above that. We think he injures his argument by denying too much; consequently, what he says fails to convince the many who have seen similar things; in fact, it drives them to the other side.

As we said, in a previous article on this subject, it has become almost a gordian knot, for the simple want of a wise discrimination as to a fact and its explanation. Persons are too much inclined to admit or deny both. The Fox girls said, "*Spirits move the tables.*" At least such is the report. Two things are here given: a fact and an explanation. The fact is, The tables move. The explanation is, "*It is done by spirits.*" But because the explanation was just such an one as two poor, simple-minded girls would give—and just such an one as educated, but superstitious minds have admitted, we have, in our strong impression of the utter absurdity of the explanation, scouted at the fact as well as at its explanation. But, as we said before, ridicule and epithet count nothing in plain argument with intelligent minds. Such want proof, not assertion.

In the above letter, it appears that Mr. Hall believes that the tables do move; that such motion is not the result of spiritual co-operation, but of an emanation, alike, possibly, to that which took place when the Saviour "*perceived that virtue had gone out of him.*" Power goes from the magnet and affects a needle at some distance from it. Who can say that a similar power may not go out of a man and affect inert matter distant from him, without his touching it? We do not say that it is so. We say that it is possible. We do not doubt that Miss Beecher rode around a room on a table, without her collusion. The point of inquiry is, "By what power?" If Mr. Hall can explain satisfactorily the seat of that power, and can go a step further and counteract it, it is certainly one of the most important additions yet made in reference to so-called "Spiritualism." The sentiment of many is, "*It may be the spirits.*" They fear to admit. They will not deny. They see spiritualists bringing forward bushels of reported facts, and they hear wise men denouncing both the facts and the reporters of them, and between their doubt of the facts and the emptiness of the denunciations, they remain in a state of indefiniteness, indecision and uncertainty of opinion most annoying. If Mr. Hall can show us where the power lies, can put the wand in our hands, and enable us to exercise it or counteract it, that is, give us the perfect control of it, then has he gained an enviable immortality, has accomplished a work well worthy of a lifetime of effort. We shall be agreeably disappointed if the Rev. gentlemen does do what his friends claim he can do. The free interpretation of the ugly word, *Volaurology*, in its connection with Spiritualism, is "Something about an emanation in connection with the will." Spiritualists are of two classes: those who are sincere in their belief, whom we pity; those who are deliberate imposters, whom we despise: to both Mr. Hall will be a benefactor if he can do what is claimed for him, because he will make the latter ashamed of themselves, and for the former, he will effect a happy deliverance.

